# Quality Prediction and Abnormal Processing Parameter Identification in Polypropylene Fiber Melt Spinning Using Artificial Intelligence Machine Learning and Deep Learning Algorithms

**DOI:** 10.3390/polym14132739

**Published:** 2022-07-04

**Authors:** Amit Kumar Gope, Yu-Shu Liao, Chung-Feng Jeffrey Kuo

**Affiliations:** Department of Materials Science and Engineering, National Taiwan University of Science and Technology, Taipei 10607, Taiwan; m10904822@mail.ntust.edu.tw (A.K.G.); jeffrey6349754@gmail.com (Y.-S.L.)

**Keywords:** melt spinning machine, artificial intelligence, machine learning, random forest, deep learning, neural network

## Abstract

Melt spinning machines must be set up according to the process parameters that result in the best end product quality. In this study, artificial intelligence algorithms were employed to create a system that detects abnormal processing parameters and suggests strategies to improve quality. Polypropylene (PP) was selected as the experimental material, and the quality achieved by adjusting the melt spinning machine’s processing parameter settings was used as the basis for judgement. The processing parameters included screw temperature, gear pump temperature, die head temperature, screw speed, gear pump speed, and take-up speed as the six control factors. The four quality characteristics included fineness, breaking strength, elongation at break, and elastic energy modulus. In the first part of our study, we applied fast deep-learning characteristic grid calculations on a 440-item historical data set to train a deep learning neural network and determine methods for multi-quality optimization. In the second part, with the best processing parameters as a benchmark, and given abnormal quality data derived from processing parameter settings deviating from these optimal values, several machine learning and deep learning methods were compared in their ability to find the settings responsible for the abnormal data, which was randomly split into a 210-item training data set and a 210-item verification data set. The random forest method proved to be the best at identifying responsible parameter settings, with accuracy rates of single and double identification classifications together of 100%, for single factor classification of 98.3%, and for double factor classification of 96.0%, thereby confirming that the diagnostic method proposed in this study can effectively predict product abnormality and find the parameter settings responsible for product abnormality.

## 1. Introduction

Modern synthetic fibers are mainly manufactured by three methods: dry spinning, wet spinning, or melt spinning. Among them, melt spinning is the most commonly used in the industry due to its low cost and the stability of the process [1,2]. However, due to the wear and tear of equipment and the resulting maintenance or replacement of different parts of the melt spinning process, product quality can be expected to deviate from the original. Inability to achieve the preset best quality and abnormality of the process may also be due to a number of other factors, such as the experience of the operator of the machine and parameter settings, among other issues. Because of the above factors, the causes of abnormality are also more difficult to analyze. Currently, the industry almost completely relies on the expertise of technologists to solve the problem.

If a specialized analysis technique can be developed to explore the link between abnormal quality and processing parameters, it will significantly enhance the maintenance of product quality, reduce abnormal analysis downtime, and stabilize process control. In order to accomplish these goals, we present a set of techniques in this work that engineers may use as criteria to determine whether product quality is declining and to quickly identify the causes of it, enhancing product quality and lowering manufacturing costs. Therefore, the goal of this study is to investigate the best melt spinning machine process parameter settings and to pinpoint those that lead to improper processing. Since PP fiber is currently one of the three major synthetic fibers that are mostly used in the production of knitwear and plush items and are produced by melt spinning, this study selects PP as the experimental material and samples from melt spinning machines as the research object. A deep learning neural network [3] is used to find the optimal processing parameters determinant of multiple quality characteristics and, in the case of quality changes, to indicate which processing parameter changes are responsible for the abnormal quality and thus solve the problem. We hope to provide the industry with a good and efficient diagnostic method for the analysis of quality abnormalities.

The selection of machining parameters in the industry is based on the experience and intuition of the engineer responsible for the machine. However, the trial-and-error process adopted is error-prone and time-consuming, so it is not optimal for complex manufacturing processes [3,4]. Chen et al. [5] used Taguchi parametric design methods combined with neural networks to optimize processing parameter settings for plastic injection molding. The experimental results showed that their neural network model not only effectively reduces the time taken for optimal processing parameter setting, but also results in more reliable product quality. The same approach is widely used in a number of different fields, including computing, science, engineering, medicine, the environment, agriculture, mining, weather prediction, business, and even art [6]. Majumdar and Majumdar [7] compared three modeling approaches for forecasting the breaking elongation of ring spun cotton yarns (mathematical, statistical, and artificial neural network models). The main reason why neural networks can successfully predict product quality is that they have the ability to learn any nonlinear mapping between input and output, and compared with traditional regression and statistical models, the results are more definitive [8]. The larger the amount of historical data, the more accurate the neural network can become [8,9].

Artificial intelligence has recently been integrated to the analytical tools of many researchers in order to shorten experimentation times and lower costs. Although abnormality diagnosis systems have been used in various industries, they are rarely met with in polymer melt spinning processing. This is surprising, since in melt spinning, as in other production processes, artificial intelligence classifiers can count different features and classify them according to different characteristics, using decision tree, support vector machine, random forest [10], or neural network rules, among others, to detect faults.

Sugumaran and Ramachandran [11] extracted and analyzed eigenvalues through vibration signals and used a decision tree to select 3 excellent features that could identify normal and abnormal eigenvalues from 11 eigenvalues. They then input them into fuzzy classifiers, showing that they can be applied to fault diagnosis. Decision trees are used not only for fault diagnosis, but also for other diagnostic purposes, like in medicine and prediction of mechanical strength [12,13]. Zimmerman et al. [14] used a CART decision tree for analysis and found that their CART-developed test, although less accurate than PCR, had good sensitivity and good predictive performance for influenza.

The random forest method has further improved the accuracy of machine learning decision trees and has been widely used in the field of artificial intelligence classification in recent years, having great advantages over other artificial intelligence algorithms. Nafees et al. [15] integrated the linear model of random forest to create a study which aims to create modelling tools for estimating the compressive and tensile strengths of plastic concrete. It is also widely used in industrial diagnosis. Cerrada et al. [16] applied random forests and genetic algorithms to do fault diagnosis and classification of gears. With a large number of features as input, the accuracy rate was as high as 97%. Sanchez et al. [17] used a deep random forest to diagnose failures of gearboxes and compared it with other machine learning algorithms such as support vector machines and the k-nearest neighbor algorithm, confirming that the random forest was the best for their data. Beyond industry, random forest classification has also achieved very good results in many other fields, such as medical analysis [18] and biological field [19]. In the field of artificial intelligence, effective classifiers can be developed not only with random forests, but also with the aforementioned neural networks. Artificial neural networks use nonlinear activation functions and have weighted outputs from multiple neurons into deeper neuron layers, with multiple layers connected in sequence to increase learning accuracy [20]. Ali et al. [21] studying the non-stationary nonlinear characteristics of rolling bearing vibration signals, used a neural network to classify bearing defects. Their experimental results showed that this method can reliably classify defects.

In this study, we proposed a set of methodologies that engineers may use as criteria to determine whether product quality is deteriorating and to immediately assist them in identifying causes, ultimately increasing product quality and lowering manufacturing costs. As a result, the goal of this research is to find the best process parameter settings for melt spinning machines and to identify those that cause anomalous processing.

## 2. Methods and Materials

**Figure 1:**
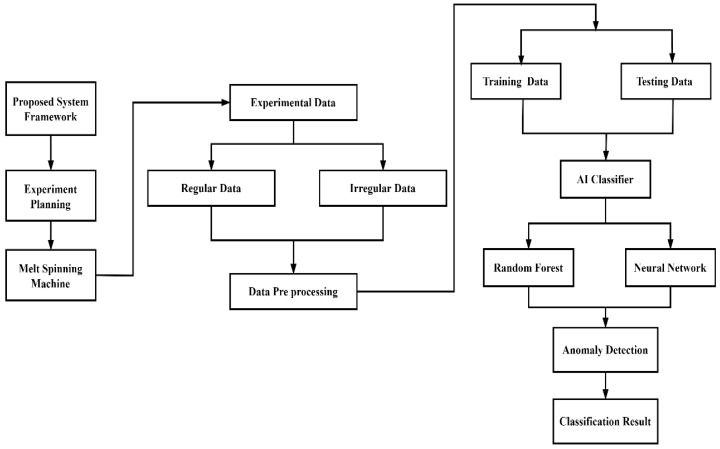
Flowchart for anomaly detection.

The experimental method and artificial intelligence steps are shown in Figure 1, including melt spinning experiment, data pre-processing, artificial intelligence classifier, single and double anomaly identification, and classification results.

### 2.1. Random Forest

The random forest is composed of decision trees, and each decision tree in the random forest is not related [22]. After obtaining the random forest, when a new input sample enters, each decision tree in the random forest makes a judgment to predict which class the sample should belong to, and finally, each decision tree votes to predict which class the sample belongs to. Although each decision tree in the random forest obtained by this algorithm is very weak, the combination of each decision tree works very well. This method is also called ensemble learning [23]. Its calculation steps:

(1) Define a random sample of size n, and randomly select n data from the data set.
(2) From the selected n data, a decision tree is trained, d features are randomly extracted for each node in the decision tree, and then the features are used to divide the node.
(3) Repeat steps 1~2 k times with improvements. The more commonly used improvement is Adaboost.
(4) Summarize the predictions of all decision trees and decide the result of this classification by voting majority or weighted voting.

AdaBoost is an algorithm that improves boosting [24] and is the most commonly used one today. The idea is to increase the weight of the samples misclassified by the previous decision trees so that each time a new decision tree is trained it can focus on training data that is easily misclassified. Each decision tree uses weighted voting instead of the average voting mechanism. Weak classifiers with higher accuracy have larger weights, and weak classifiers with lower accuracy have lower weights.

First, a set of training data is given:

(1) $$\left(\left\{\left(x_{1},y_{1}\right),\left(x_{2},y_{2}\right),\ldots ,\left(x_{n},y_{n}\right)\right\}\right)$$

Assuming that the weight of the kth time is $w_{k}^{i}$, the weight of each sample of the first decision tree classifier is:

(2) $$w_{1}^{i}=\frac{1}{n}$$

First train the first decision tree classifier $f_{k}\left(x\right)$ with weights $w_{k}^{i}.$

Assuming that L decision tree classifiers are trained, when training the kth one:

(3) $$a_{k}=0.5*\mathrm{In}(\frac{1-\varepsilon_{k}}{\varepsilon_{k}})$$

(4) $$w_{k+1}^{i}=\left\{ \begin{array}{l} w_{k}^{i}*e^{{}_{a_{k}}}\;\;if\;\;f_{k}(x_{i})\neq y_{i}\\ w_{k}^{i}*e^{{}_{-a_{k}\;}}\;\;if\;\;f_{k}(x_{i})=y_{i} \end{array}\right.$$

The $\varepsilon_{k}$ is the error of the kth decision tree classifier.

Finally, $f_{L}\left(x\right)$ decision tree classifiers are obtained, and the results of all decision tree classifiers are weighted to vote:

(5) $$H(x)=sign(\sum_{K=1}^{L}a_{k}f_{k}(x))$$

### 2.2. Neural Network

**Figure 2:**
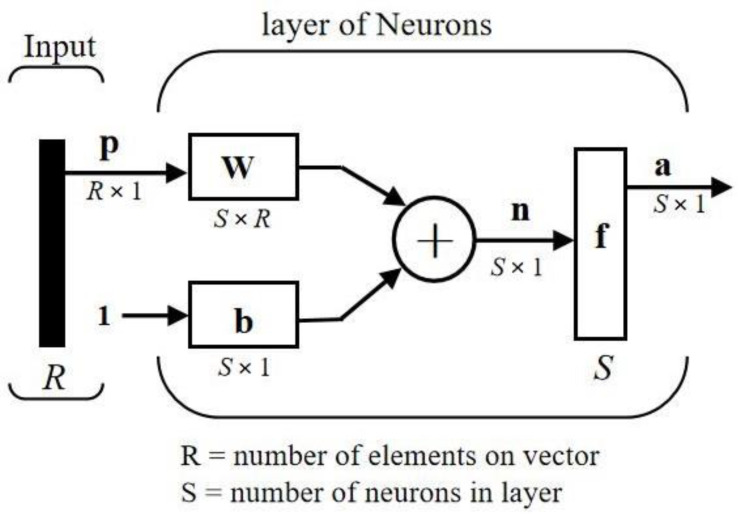
Single-layer neural network architecture.

A neural network mimics the human brain, in a structure consisting of thousands of interconnected neurons. A neuron can be connected to multiple neurons in its rear layer for output, and multiple neurons in a front layer for reception. Neurons and perceptron’s are mathematical functions that multiplies input data from the input layer (×1, ×2…) by a weight (*w*1, *w*2…) and adds a bias (*b*) to the weighted inputs (hidden layer). The result is then put via an activation function (*f*) to introduce nonlinearity to the network. All incoming data points receive a weight, are multiplied and added together, and passed to a non-linear activation function. An example of a single-layer neural network-like architecture is shown in Figure 2.

The output of each neuron:

(6) a=f(wp+b)

After calculating the loss between the output layer of the neural network and the correct value, the neural network will modify the neural network parameters through the back-propagation algorithm. Since neural networks are inherently nonlinear, consisting of multiple inputs and multiple outputs, they are suitable for modeling complex nonlinear systems.

In order to find the smallest loss function, the training of neural network often uses the gradient descent algorithm to achieve optimization.

(7) $$w_{i}=w_{i-1}-\gamma \frac{\partial L}{\partial W}$$

where *W* is the weight parameter; *γ* is the learning rate; *L* is the loss function. $\frac{\partial L}{\partial W}$ is the gradient of the loss function to the weight parameter.

### 2.3. Activation Functions

Activation functions are functions that are used in neural networks to compute the weighted total of input and biases, which is then used to determine whether or not a neuron can activate [25]. They are used to control the outputs of our neural networks in a variety of areas, including object recognition and classification, as well as other domains, to name a few, with early research findings demonstrating unequivocally that good activation function selection improves neural network computing results. ReLU, Mish, and Sigmoid Functions are the activation functions employed in this work.

### 2.4. Optimization Techniques

Optimization is one of the important aspects of deep learning, as it helps a model train better when the weights are modified, so that it can reduce the loss error and also handle the dimensionality problem during back propagation. Ruder [26] investigated the convergence time, number of fluctuations, and parameter update rate of multiple stochastic gradient descent-based optimization algorithms, including SGD and SGD with momentum, Adam, and RMSProp, using varying numbers of iterations and particular values of the test function.

### 2.5. Materials

**Figure 3:**
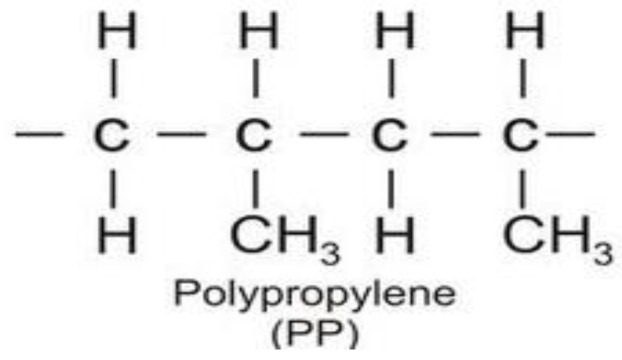
Chemical structure of PP.

PP is an outstanding textile material which is purchased from polyacrylic polymer Globalene 6331 (LCY Chemical Corp., Taipei, Taiwan). The chemical structure is shown in Figure 3. It features abrasion resistance, flexibility, high strength resistance, light weight, strong antistatic character, good chemical resistance, and a low cost. Acid and alkali resistance, water repellency, quick drying, bacteria repellency, below thermal conductivity, warmth retention, low glass transition point, low temperature resistance, low energy consumption, low CO_2_ emission, decomposability, no dyeing wastewater pollution, and oil absorption are among its advantages over PET, Nylon 6, and Nylon 66 fibers. PP fiber is now widely utilized and has gained economic relevance in the production of home furnishings and industrial uses. The PP fiber is one of three primary synthetic fibers and is generally made via melt spinning. In the vertical or horizontal screw extruder, the acrylic resin is heated and molten, then extruded by nozzle through the metering pump and cooled to fiber in the air [27,28].

## 3. Experiment Plan

**Figure 4:**
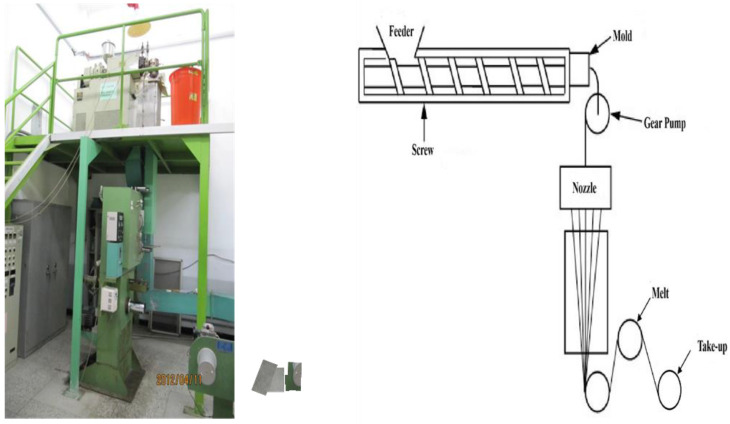
Melt spinning machine process.

In this study, a melt spinning machine was used as the research machine, and PP was used as the material. The melt spinning machine uses heating to make the material appear in a molten state. The material was then conveyed through the screw and the gear pump, so that it is continuously extruded through the spinning nozzle, and wound into a drum through the roller. Since the melt spinning machine includes feeder, screw heating zone, gear pump, spinning nozzle, and take up system, as shown in Figure 4, the screw temperature, gear pump temperature, die head temperature, screw speed, gear pump speed, and take-up speed are designed as process parameters to discuss the quality of the fiber process. At the same time, a group of neural networks was trained using historical experimental data to predict the multiple characteristics of quality on the basis of various processing parameter values.

Half the quality characteristics obtained were used as input feature values for the training of a quality abnormality classifier. After training, the other half were input as test samples to confirm whether the classifier could identify processing parameters responsible for the quality abnormality. Assuming the identification was successful, the diagnosis system for the melt spinning machine was complete.

### 3.1. Materials Analysis

**Figure 5:**
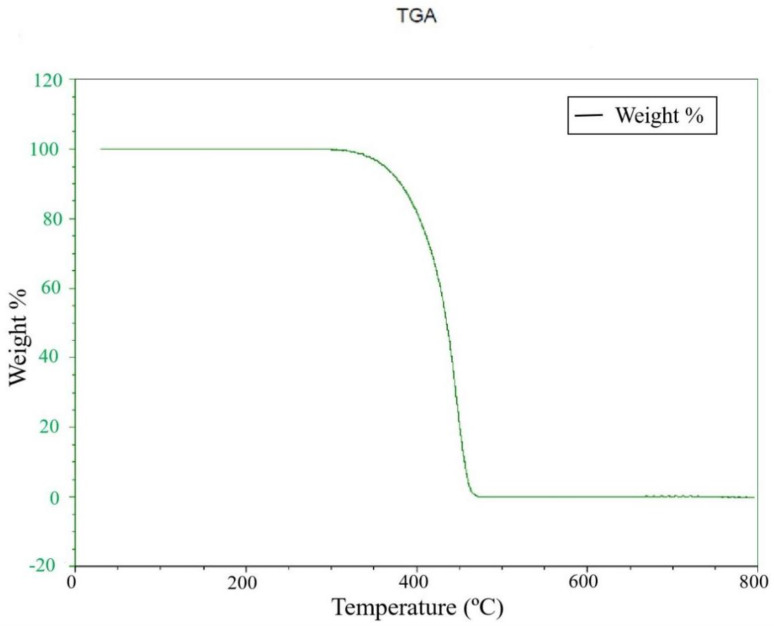
Thermogravimetric analysis of PP.

**Figure 6:**
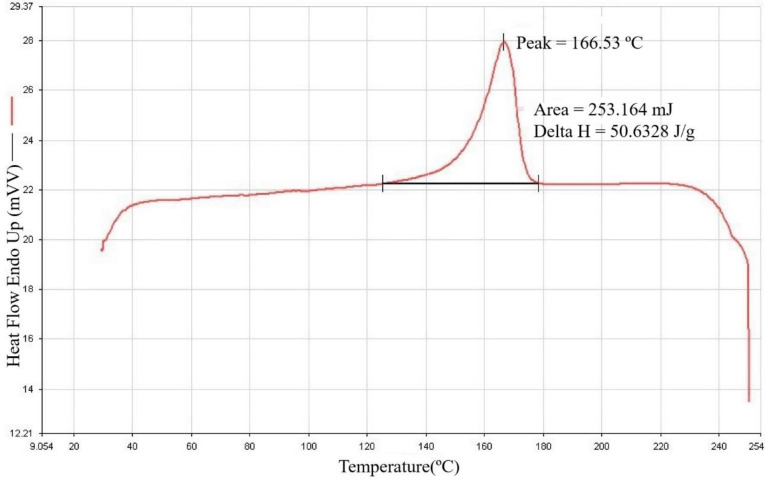
DSC analysis of PP.

PP was selected as the experimental material in the study because of its characteristics, namely, easy processing, mechanical strength, strong elasticity, resistance to staining, lightness, and low price. Before the experiment, it was necessary to find its melting point and thermal cracking point to plan settings for the machine. To learn the temperature of the thermal cracking point, a thermogravimetric analyzer was used, with a thermal differential analyzer used to measure its melting point. A thermogravimetric analysis diagram and differential scanning calorimetry (DSC) diagram are shown in Figure 5 and Figure 6. It can be seen that the thermal cracking point of PP is about 400 °C, so this temperature was not exceeded during the experiment, as it risked contaminating the machine. The melting point is about 166 °C. Therefore, the temperature was kept above this level during the experiment. Temperatures lower than this temperature also risked damaging the machine.

### 3.2. Multi-Quality Characteristic Prediction

#### 3.2.1. Experimental Data

A total of 440 historical melt spinning measurement records were used as data in the study. There were six processing parameters, namely, screw temperature, gear pump temperature, die head temperature, screw speed, gear pump speed, and take-up speed. The corresponding quality characteristics were fineness, breaking strength, elongation at break, and modulus of resilience. These 440 samples were randomly split into a 330-item training data set and a 110-item validation data set, using the k-fold cross-validation method to produce the best model for subsequent analysis when the model was finally optimized.

#### 3.2.2. Data Processing

Due to the different units of measurement of all the input independent variables, the output dependent variables, and the different value size characteristics, comparability was impaired. To solve this problem, the data was first normalized for this experiment. The range of processing parameters of the original independent variables is shown in Table 1.

#### 3.2.3. Neural Network Training

A neural network was used for the prediction of the multiple quality characteristics. The input variables were the processing parameters of the melt spinning machine: screw temperature, gear pump temperature, die head temperature, screw speed, gear pump speed, and take-up speed. The output variables were the corresponding quality characteristics: fineness, breaking strength, elongation at break, and modulus of resilience.

**Figure 7:**
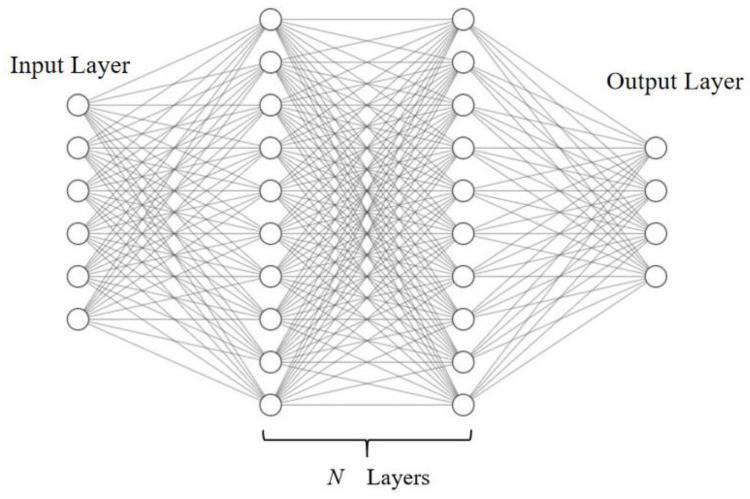
The architecture of the neural network.

The architecture of the neural network is shown in Figure 7. The number of hidden layers and the number of neurons in each layer of the neural network were variables used to find the best results using the grid search method.

**Figure 8:**
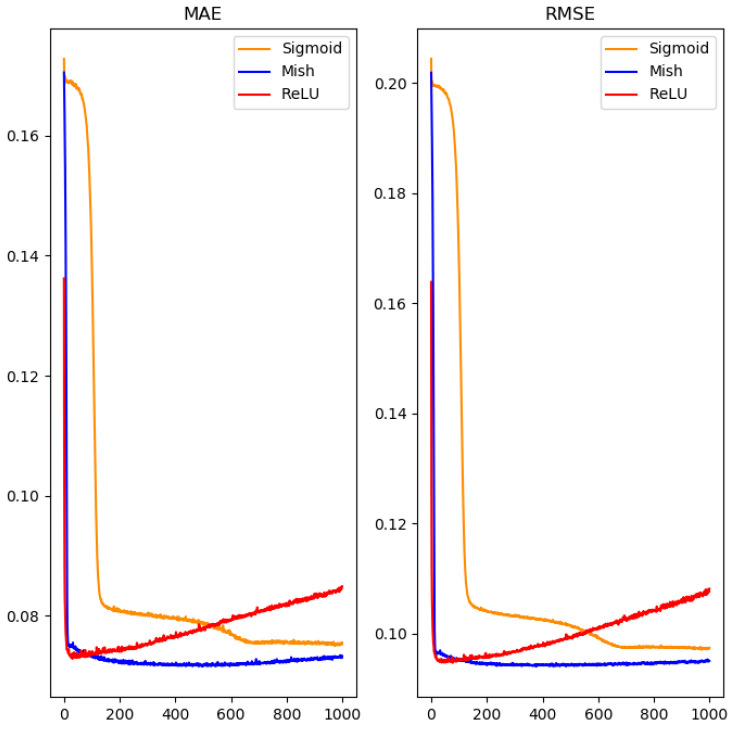
The effect of common activation functions used in the hidden layer on the performance.

In order to avoid the problem of over-fitting in neural networks, this study added the dropout method between hidden layers for regularization. However, the experimental results showed that the use of dropout regularization in the neural network only slightly improved the prediction of values. In order to normalize output values in the range 0 to 1, the output layer adopted the sigmoid function as shown in Equation (8). The activation function of the remaining layers was different from the commonly-used ReLU activation function [29]. The novel Mish function was used instead [30], as shown in Equation (9). The experimental process is shown in Figure 8.

(8) $$sigmoid(x)=\frac{1}{1+e^{-x}}$$

(9) $$Mish(x)=x\tanh(\ln(1+e^{x}))$$

As can be seen from Figure 8, the Mish function behaves differently than the commonly-used ReLU and sigmoid functions [31]. It can effectively and quickly converge the loss, and it is less prone to the problem of network weakening when the loss increases in a long iteration.

#### 3.2.4. Evaluation Criteria and Training Results

In order to evaluate the performance of the neural network, the study adopted the commonly-used statistical standards, mean absolute error (MAE) and root mean squared error (RMSE), as evaluation methods. The formulas are shown in Equations (10) and (11). The lower the value, the better the performance of the neural network.

(10) $$MAE=\frac{1}{n}\sum_{i=1}^{N}\left|\frac{{\hat{y}}_{i}-y_{i}}{y_{i}}\right|$$

(11) $$RMSE=\sum_{i=1}^{N}\sqrt{{\left({\hat{y}}_{i}-y_{i}\right)}^{2}/N}$$

where *N* represents the quality characteristic quantity, and ${\hat{y}}_{i}$ and $y_{i}$ represent the predicted value and the actual value, respectively.

The mean absolute error and root mean square error of the training results for the validation data sets with neural network grid search are shown in Table 2 and Table 3, respectively.

**Figure 9:**
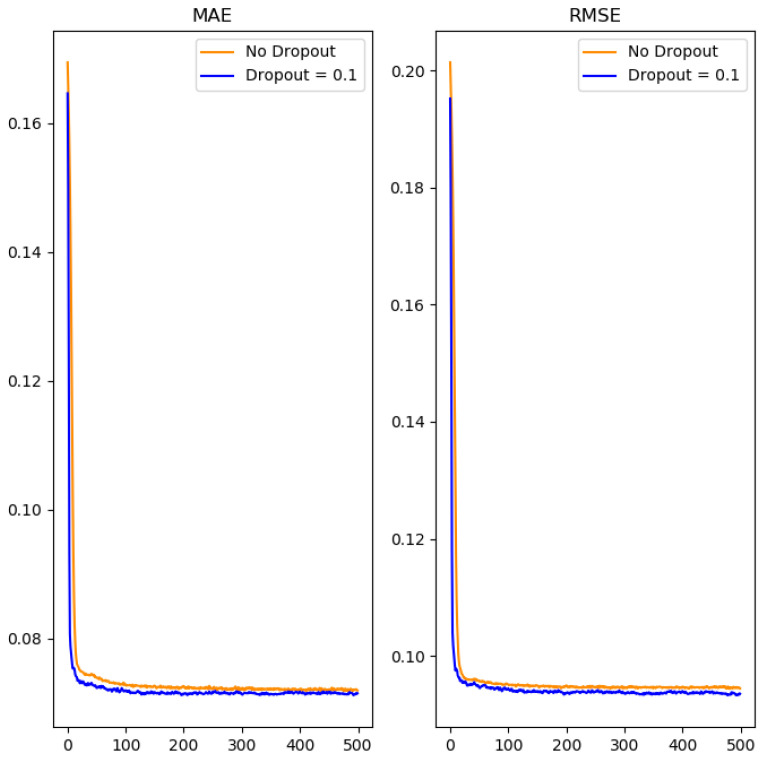
The effect of adding dropout between hidden layers on performance.

It is observed from Table 2 and Table 3 that the mean absolute error and the root mean square error can be reduced by increasing the number of neurons and the hidden layers. However, if the number of these two is increased too much, it will lead to overfitting, so that the error in the validation dataset increases. After using the grid search method [32] to obtain the optimal number of hidden layers and neurons in the neural network, we added dropout regularization between the hidden layers in the neural network to reduce the problem of overfitting, as shown in Figure 9.

Despite trying to reduce overfitting in the neural network through the use of dropout regularization, the experimental results showed it only slightly reduced errors in predicted numerical values in the validation data set. Then, we used three algorithms to improve gradient descent for the final optimization of the neural network, namely SGDM [33], RMSProp [34], and Adam [35]. In order to solve the common problem of the basic gradient descent falling into a local optimal solution and not being able to escape, we made use of the SGDM gradient descent method with momentum.

(12) $$V_{i}=\beta V_{i-1}-\gamma \frac{\partial L}{\partial W}$$

(13) $$W_{i}=W_{i-1}+V_{i}$$

The above equation is the SGDM gradient descent formula with momentum. Compared with the basic SGDM gradient descent method, directional velocity *V* and momentum *β* are added.

**Figure 10:**
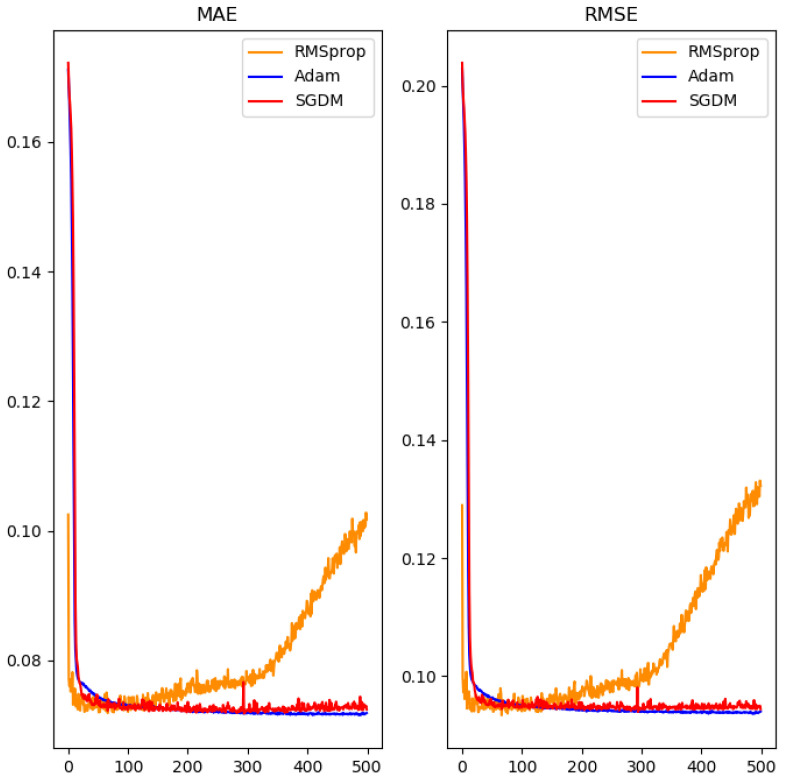
Neural network using different gradient descent methods for comparison.

**Figure 11:**
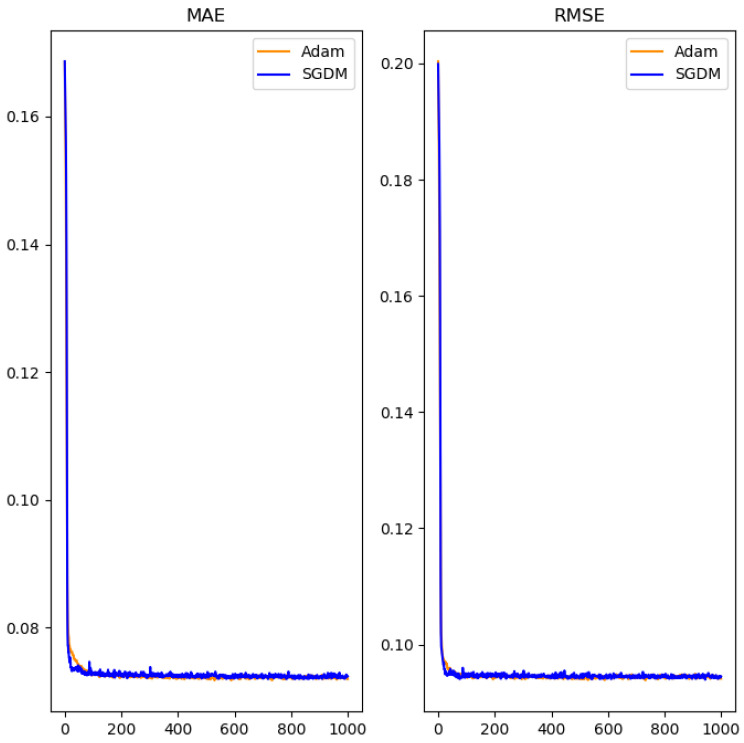
The comparison of Adam and SGDM algorithm.

Starting by testing with a smaller number of iterations, as shown in Figure 10, we found that the RMSProp algorithm reduced error rapidly at the beginning, but its performance declined after a larger number of iterations. The Adam algorithm and the SGDM algorithm, on the other hand, behaved similarly and converged effectively on a smaller error rate. Therefore, the number of iterations was extended in the experiment, and only the performance of the Adam algorithm and the SGDM algorithm was compared. The results are shown in Figure 11.

After 1000 iterations, the Adam algorithm and the SGDM algorithm had quite similar training curves, with mean absolute errors of 0.0713 and 0.0709, and root mean square errors of 0.0917 and 0.0905, respectively. Although there was not much difference between the two, the SGDM algorithm reduced the loss slightly more effectively than the Adam algorithm. The detailed optimal training process is shown in Table 4.

Finally, the quality characteristic values predicted by the neural network are shown in Table 5 as a figure between 0 and 1 calculated by the sigmoid function. The results show that the neural network effectively predicted the multiple quality characteristic values that particular processing parameter settings would produce. Not only could expected results be predicted before an experimental run, but the search for optimization parameters could also be further carried out.

**Figure 12:**
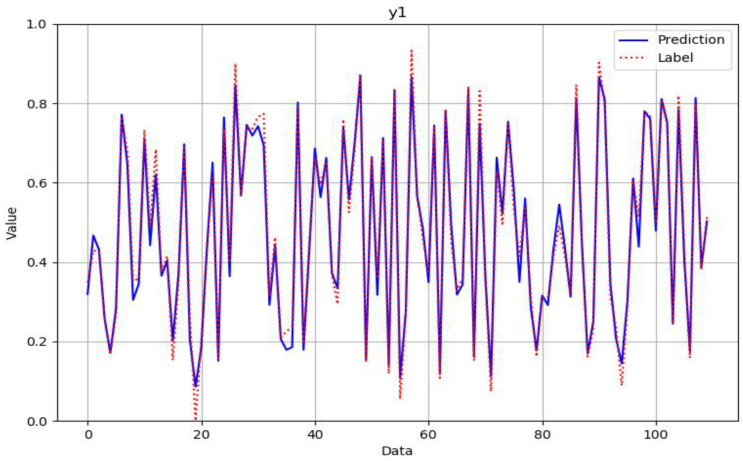
Comparison of predicted and actual values in the fineness verification data set after normalization.

**Figure 13:**
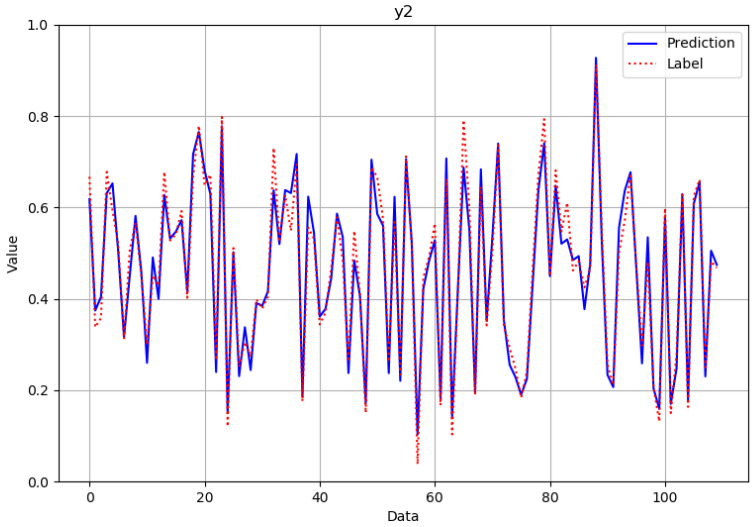
Comparison of predicted and actual values in the fracture strength verification data set after normalization.

**Figure 14:**
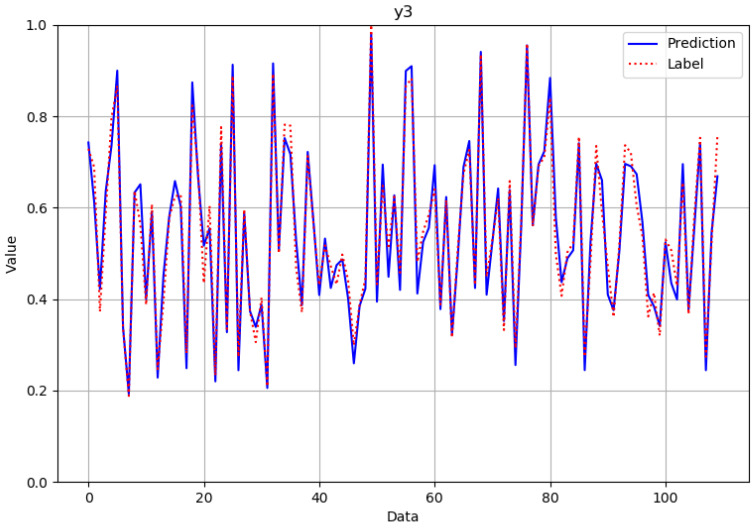
Comparison of predicted and actual values in the elongation at break verification data set after normalization.

**Figure 15:**
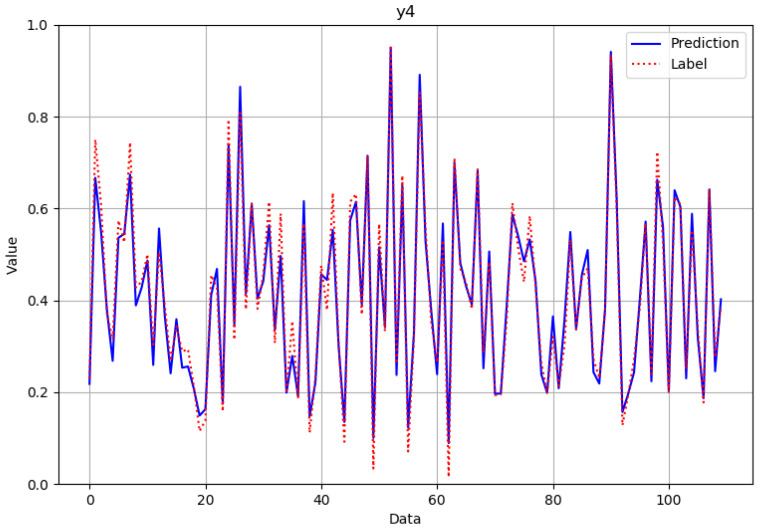
Comparison of predicted and actual values in the modulus of resilience verification data set after normalization.

As can be seen from Figure 12, Figure 13, Figure 14 and Figure 15, the neural network model could successfully and effectively predict the effect of various combinations of processing parameters on the corresponding fineness, breaking strength, elongation at break, and modulus of resilience quality characteristics.

Compared with the traditional Taguchi analysis method, which requires the setting up of orthogonal arrays, carrying out main effect analysis, analysis of variance, confirmatory tests, and undertaking other time-consuming steps [5], the neural network model conducts self-training and learning with past historical data, meaning it can analyze the data more efficiently.

It was the aim of this study to obtain optimal processing parameters for minimum fineness and maximum breaking strength, elongation at break, and modulus of resilience. Therefore, because of the high speed at which deep learning neural networks could be calculated (one data prediction does not require one millisecond), the grid search method was used to exhaustively find the best combination of processing parameters with a mean square error minimum for fineness and a maximum one for breaking strength, elongation at break and modulus of resilience. The result of the search was that when the screw temperature is 180 °C, the gear pump temperature is 220 °C, the die head temperature is 240 °C, the screw speed is 7.5 rpm, the gear pump speed is 15 rpm, and the take-up speed is 700 rpm, the output quality characteristic is that the fineness is 243 Denier (D), the breaking strength is 3.4 N/mm^2^, the breaking elongation is 643%, and the elastic energy modulus is 9.13 N/mm^2^, as shown in Table 6.

### 3.3. Creating Historical Data and Abnormal Samples

Having obtained the best parameters with the neural network grid search method, this set of processing parameters was used as experimental parameters to produce one data set made up of 20 normal samples, as shown in Table 7. Then, to produce two more abnormal experimental data sets, for the first with one abnormal parameter setting and the second with two abnormal settings, one or two processing parameters were changed in sequence, as shown in Table 8. Therefore, for the first single-factor abnormal samples for the A setting there, the value was Abnormal 1 and for the second samples it was Abnormal 2, with the remaining processing parameters staying the same. For two-factor abnormal samples, two processing parameters were changed at a time, with the others staying the same. Therefore, for ones where the A and B settings were changed, at the same time, they were Abnormal 1 and then they were Abnormal 2. The remaining processing parameters were changed according to this same rule.

In this study, for both normal and abnormal data sets, the generation of 20 samples was taken as a standard, so a total of 20 samples were produced for the best parameters, There were twenty samples for each single-factor abnormal processing parameter (both Abnormal 1 and Abnormal 2), and 20 samples for each two-factor abnormal sample pairing (both Abnormal 1 and Abnormal 2). A total of 20 normal samples, 120 (=6 × 20) single-factor abnormal samples, and 300 (=15 × 20) two-factor abnormal samples were obtained.

### 3.4. Abnormal Processing Parameter Classifier Model Training

In order to determine which processing parameters cause quality characteristic abnormalities, and to improve the process yield of the melt spinning machine, the 420 samples of the abnormal data sets and 20 of the normal data set were applied to train an artificial intelligence classifier, using the neural network to generate quality characteristic predictions. Input feature $x_{i}$, as shown in Equation (14), was the differences between the four quality characteristics $y_{i}$ (fineness, breaking strength, elongation at break, elastic energy modulus) actually obtained in the abnormal sample data and the corresponding ${\hat{y}}_{i}$ predictions of the neural network quantity, as shown in Table 9. The output was one-hot encoded classification results for the processing parameters of screw temperature, gear pump temperature, die head temperature, screw speed, gear pump speed, and take-up speed, as shown in Table 10.

(14) $$x_{i}=({\hat{y}}_{i}-y_{i})x$$

When training the classifier, if all the data are directly classified as the result of one or two factors, errors are likely because some values are too close to allow abnormal processing parameter settings to be correctly judged. In order to improve the accuracy of the classifier, it was necessary to divide the abnormal samples into one-factor or two-factor groups first, and then treat them separately. Therefore, this study needed to use a total of three classifiers to make predictions. When the classifier was classified as single or double, the output was a single or two-factor abnormality; when a single-factor classification was used, the output was the corresponding abnormal processing parameter type; when a two-factor classification was used, the output was the corresponding two abnormal processing parameter types.

**Figure 16:**
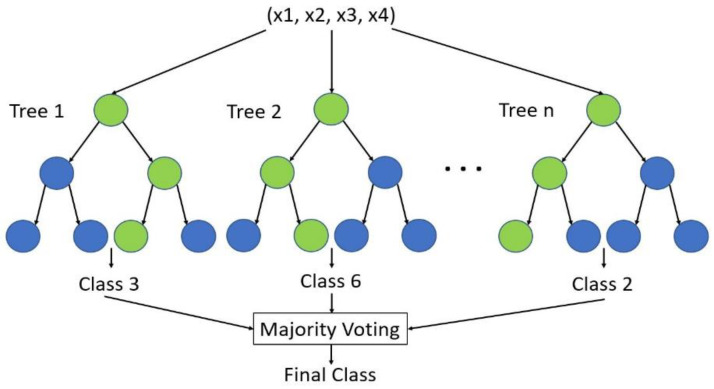
Random forest architecture.

Because relevant studies in the literature have achieved outstanding performance with a number of different classifiers, this study compared these commonly-used machine learning methods with the results found here. Classification methods such as decision trees, random forests, support vector machines, and neural network methods such as deep learning are all possible bases for comparison. Since each of these classifier methods have many hyper parameters to be adjusted, the grid search method was used to find the best parameter combination of each method. A random forest architecture diagram is shown in Figure 16.

#### 3.4.1. Single and Double Identification

**Figure 17:**
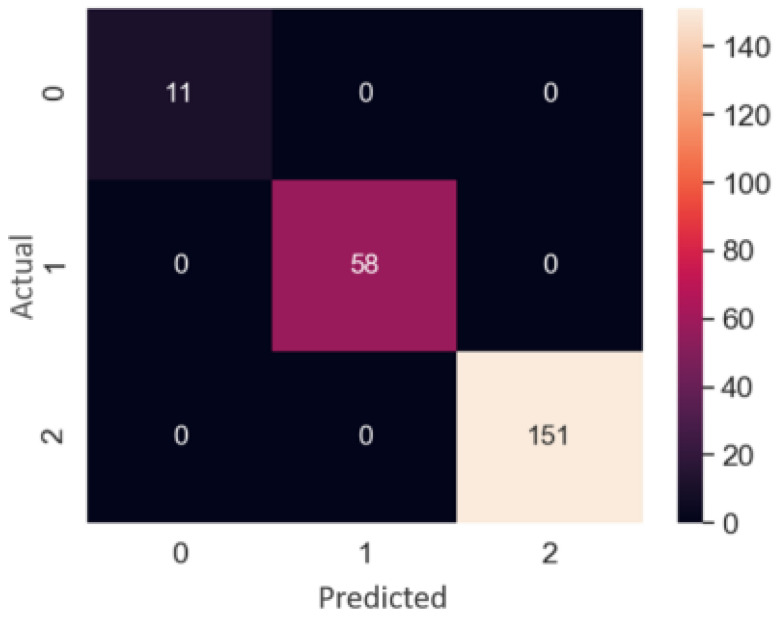
The confusion matrix of the classification results of random forest single and double identification.

For determining the difference for each sample between the actual four quality characteristics of the abnormal sample data, and the four quality characteristics predicted by the neural network quantity, an array of 4 values was used as the input feature of the classifier. There were 420 abnormal samples and 20 normal samples in the data set. The data set was randomly divided into a 220-item training data set and a 220-item verification data set. First, the training data set was used to construct a model, and then the verification data set was used for testing. The purpose of the verification data set was to determine whether the melt spinning abnormal diagnosis system could adequately detect processing parameters responsible for abnormalities or not. Training also used grid search and cross-validation to find the best settings for each classifier. Evaluation of the classifier used the detection success rate, which is the ratio of the number of samples correctly identified to the total number of samples in all the validation data sets, as shown in Equation (15). The larger the value, the better. The detection success rates of various classification methods are shown in Table 11. The success of the random forest classification method can be seen in the confusion matrix in Figure 17, where 0 means no abnormality, 1 means a single abnormality, and 2 means a double abnormality. In the validation data set, the method was the best at identifying single and double abnormalities, and there were no lack of abnormalities, single abnormalities, and double abnormalities in the 220 samples which it missed. All predictions were correct. As for grid search hyperparameters, a random forest with eight decision trees and a maximum depth of four worked best.

(15) $$\begin{array}{l} \mathrm{Detection}\;\mathrm{success}\;\mathrm{rate}=\frac{\mathrm{Correct}\;\mathrm{number}\;\mathrm{of}\;\mathrm{samples}\;\mathrm{to}\;\mathrm{be}\;\mathrm{tested}}{\mathrm{All}\;\mathrm{sample}\;\mathrm{numbers}} \end{array}$$

#### 3.4.2. One-Factor Classification

**Figure 18:**
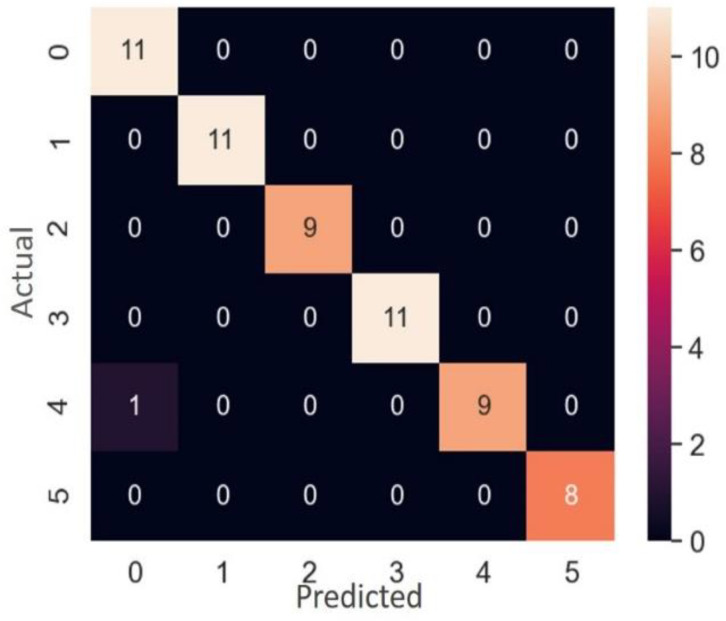
Confusion matrix of random forest single-factor classification results.

Single-factor abnormal discrimination can be carried out after single-double identification is performed and has indicated there is one abnormal parameter setting. In development of the study’s classifier, there were a total of 120 samples, 60 were randomly selected for training, and the other 60 formed the verification data set. The results for single-factor classification, shown in Table 12, were similar to the single-double identification ones, and the use of random forest classification was again the best. From the confusion matrix in Figure 18, where 0 to 5 represent screw temperature, gear pump temperature, die head temperature, screw speed, gear pump speed, and take-up speed, respectively, it can be seen that only one gear pump speed abnormality in the 60-item verification data set was misjudged as an abnormal screw temperature, so the detection success rate was as high as 98.3%. Identification by the random forest classifier of which processing parameter caused the abnormality worked very well. As for grid search hyperparameters, the random forest with twelve decision trees and a maximum depth of four performed best.

#### 3.4.3. Two-Factor Classification

**Figure 19:**
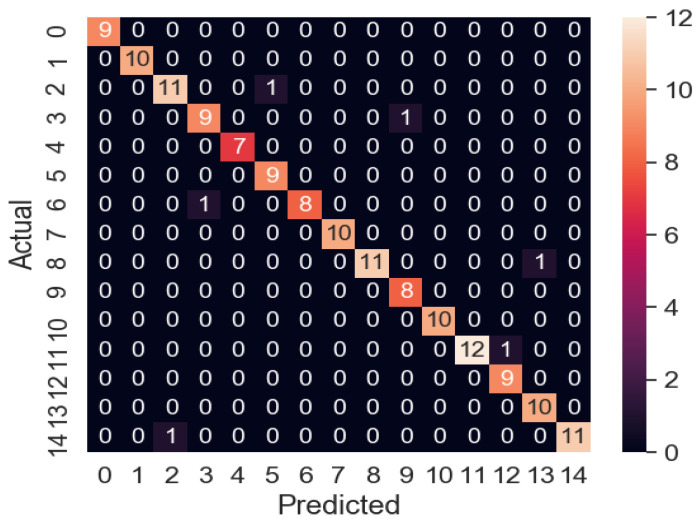
Confusion matrix of random forest two-factor classification results.

Finally, a two-factor abnormality classifier was developed with a total of 300 samples, 150 randomly selected for the training data set and 150 for the verification data set. The experimental results are shown in Table 13. It can be seen that the random forest classifier performed much better than the decision tree and neural network ones. Its confusion matrix is shown in Figure 19, with 0 to 4 representing the combination of screw temperature and the remaining 5 abnormal processing parameters, 5 to 8 representing the combination of gear pump temperature and the remaining 4 abnormal processing parameters, 9 to 11 representing the combination of die temperature and the other three abnormal processing parameters, 12 and 13 representing the combination of the screw speed and the other two abnormal processing parameters, and 14 representing the abnormal processing parameter combination of the gear pump speed and the take-up speed. It can be observed that only 6 misjudgments were made of verification data set samples, and the detection success rate was as high as 96.0%. These 6 misjudged samples were made up of a combination of different abnormal processing parameters, with no parameter appearing in more than one sample, indicating that model overfitting is not a problem. As for the grid search hyperparameters, the random forest with twenty-four decision trees and a maximum depth of six worked best.

It can be concluded that the random forest achieved the best accuracy rate, with results similar to those in the literature. With comparatively few data samples, it also exhibited greater anti-overfitting resistance. This study’s random forest classifier was compared with the use of the decision tree classifier and the four values obtained by the RAM method as input features in their ability to identify abnormal processing parameters. As can be seen from the final overall accuracy rate in Table 14, the results for both are outstanding. However, using the deep learning neural network and random forest classifier of this study to do abnormal processing parameter detection avoids the need to compute Hotelling’s T^2^ for abnormal product detection first, by directly classifying the data on the basis of the results obtained from the deep learning neural network. To use the decision tree with the RAM method too much process calculation is required [36,37]. For example, supposing there is some bias in the calculation of Hotelling’s T^2^ at the beginning. This will lead to indirect errors in the RAM method and the feature input of the decision tree, resulting in misjudgment of the final detection result. In addition, the calculation time required for the final abnormal processing parameter detection in this study is only 0.08 s, meaning it is more efficient in comparison.

## 4. Conclusions

This study applied a deep learning neural network and random forest from machine learning in artificial intelligence to the optimal quality prediction of multiple quality parameters and quality abnormality diagnosis of melt spinning machines. It included six processing parameters and four qualities. The conclusions are as follows.

(1) The deep learning neural network is used for experiments, 440 pieces of historical data are trained, and multiple quality optimization parameters are searched by using the characteristic grid of deep learning rapid calculation. The deep learning neural network was used to generate quality predictions, trained on a 440-item historical data set, and multiple quality optimization parameters were searched for using rapid deep-learning characteristic grid calculations. Compared with the traditional Taguchi analysis method, the neural network model conducts self-training and learning using past historical data, which means the research can proceed faster, analysis is more efficient, and conclusions are more robust, because a calculation error in one step will not affect the overall detection system.
(2) This research compared several artificial intelligence machines learning and deep learning classifiers that have obtained outstanding results in the related literature, and finally selected the random forest as being the best, because its classifier belongs to ensemble learning, and the classifier is resistant to overfitting. Its ability to detect the cause of quality problems was better than that of other classifiers. As an indication the success rate of single and double identification was 100%, the success rate of single factor classification was 98.3%, and the success rate of double factor classification was 96.0%. It can be seen that the proposed method offers an effective way to identify the problematic machine settings, causing problems in quality control after the engineer has measurements of the abnormality so that the settings can be quickly modified to improve production yield.
(3) This study applied the methods of artificial intelligence to the development of an abnormal processing PP fiber melt spinning parameter identification system which can quickly find abnormal settings and reduce unnecessary cost and waste. In the future, different online detection systems matching the capabilities of this system for various other kinds of material will be added to the resources available to production engineers seeking to apply the developed identification system for its functions of selection and evaluation.

## Figures and Tables

**Table 1:** The range of melt spinning machine processing parameters.

Range	Screw Temperature	Gear Pump Temperature	Die Head Temperature	Screw Speed	Gear Pump Speed	Take-Up Speed
Lowest	160 °C	200 °C	210 °C	5 rpm	15 rpm	300 rpm
Highest	200 °C	240 °C	250 °C	10 rpm	25 rpm	700 rpm

**Table 2:** Mean absolute error of validation data sets for neural network grid search.

	Neurons in Each Layer	20	30	40	50
No. of Hidden Layers					
2		0.084	0.079	0.088	0.085
3		0.078	0.078	0.079	0.080
4		0.076	0.074	0.073	0.074
5		0.075	0.075	0.075	0.077

**Table 3:** Root mean square error of validation data sets for neural network grid search.

	Neurons in Each Layer	20	30	40	50
No. of Hidden Layers					
2		0.104	0.102	0.105	0.104
3		0.101	0.101	0.102	0.102
4		0.098	0.095	0.093	0.096
5		0.097	0.097	0.098	0.101

**Table 4:** Best training results of neural network classes.

Basic Neural Network	ReLU	Mish	Dropout	Adam	RMSProp	SGDM	MAE	RMSE
T	T			T			0.075	0.097
T		T		T			0.073	0.091
T		T	T	T			0.071	0.092
T		T	T		T		0.072	0.093
T		T	T			T	0.071	0.091

(Notes: T stands for use).

**Table 5:** The outputs of the neural network.

	Quality	Fineness	Breaking Strength	Elongation at Break	Modulus of Resilience
No.					
1		0.3756	0.4750	0.7792	0.3485
2		0.4911	0.6983	0.1402	0.9094
3		0.6038	0.1909	0.6664	0.8443
4		0.7908	0.1200	1	0.7692
5		0.1688	0.3887	0	0.5886
6		0.7128	1	0.2288	0.1552
7		0.6479	0.5397	0.0466	0.7645
8		0.4227	0	0.7121	0.5876
9		0.5020	0.6814	0.5338	0.6714
10		0.1160	0.4072	0.6850	1
11		0.4236	0.5923	0.7031	0
12		0.9014	0.5112	0.4281	0.9307
13		0.1014	0.8892	0.8902	0.9345
14		0.7915	0.7402	0.6798	0.4314
15		0	0.2648	0.1486	0.4834
16		0.4965	0.3988	0.7897	0.6255
17		1	0.6118	0.5782	0.6722
18		0.1556	0.5548	0.5080	0.5609
19		0.1875	0.9408	0.7626	0.8439
20		0.0891	0.8904	0.8377	0.9105

**Table 6:** Predicted quality data for optimal processing parameters.

	Fineness (dB)	Breaking Strength (dB)	Elongation at Break (dB)	Modulus of Resilience (dB)
Predication value	0.183	0.872	0.947	0.935
Denormalized value	243	3.4	643	9.13

**Table 7:** Twenty data samples with the best parameters.

Best Parameter Data					
	Quality	Fineness (Diner)	Breaking Strength (N/mm^2^)	Elongation at Break (%)	Modulus of Resilience (N/mm^2^)
Samples					
1		236	3.1	641.972	9.03
2		237	2.8	648.305	9.40
3		237	3.4	648.357	9.39
4		231	2.8	644.224	9.28
5		241	3	648.265	9.45
6		249	3.6	635.923	9.52
7		227	2.9	642.845	8.74
8		231	3.6	641.218	8.82
9		232	3.6	646.801	9.30
10		238	3.5	645.216	9.36
11		241	3.5	643.506	9.03
12		231	3.5	640.725	9.48
13		236	3	641.378	9.03
14		247	3.4	646.776	9.49
15		251	2.8	643.393	9.79
16		245	3.5	642.942	8.99
17		240	2.7	641.155	9.54
18		240	3.6	642.811	9.20
19		234	3.6	640.911	9.04
20		257	3.6	646.926	8.87

**Table 8:** Abnormal sample processing parameter settings.

	A	B	C	D	E	F
	Screw Temperature (°C)	Gear Pump Temperature (°C)	Die Head Temperature (°C)	Screw Speed (rpm)	Gear Pump Speed (rpm)	Take-Up Speed (rpm)
Normal	180	220	240	7.5	15	700
Abnormal 1	190	200	220	5	20	300
Abnormal 2	200	210	230	10	25	500

**Table 9:** Quality data for 10 abnormal samples.

	Quality	Fineness (dB)	Breaking Strength (dB)	Elongation at Break (dB)	Modulus of Resilience (dB)
Sets					
1		224	2	643.791	8.97
2		249	2.8	655.667	7.66
3		562	1.8	591.197	9.14
4		598	2.9	642.068	6.99
5		316	2.2	520.831	8.92
6		283	3.3	531.791	6.09
7		551	2.8	645.044	8.73
8		347	3.1	647.541	8.96
9		254	3.2	606.269	9.60
10		296	2.2	638.988	8.93

**Table 10:** Classification results for the corresponding abnormal samples of Table 9.

	Processing Parameter	Screw Temperature	Gear Pump Temperature	Die Head Temperature	Screw Speed	Gear Pump Speed	Take-Up Speed
Sets							
1		0	1	0	0	0	0
2		1	0	0	1	0	0
3		0	0	0	1	1	0
4		0	0	0	0	0	1
5		0	1	0	0	1	0
6		1	0	0	0	0	1
7		0	0	1	0	0	0
8		0	0	0	1	0	0
9		1	0	0	0	0	0
10		0	1	1	0	0	0

**Table 11:** Comparison of single and double identification and classification methods.

Method	Single and Double Identification Detection Success Rate
Decision tree	98.5%
Radom forest	100%
Support vector machine	98.1%
Neural network	98.1%

**Table 12:** Comparison of single factor classification methods.

Method	Single Factor Classification Detection Success Rate
Decision tree	95.0 %
Radom forest	98.3 %
Neural network	96.8 %

**Table 13:** Comparison of two-factor classification results.

Method	Two-Factor Classification Detection Success Rate
Decision tree	91.8%
Radom forest	96.0%
Neural network	89.3%

**Table 14:** Overall classification accuracy comparison.

		Single and Double Identification	One-Factor Classification	Two-Factor Classification
	
This research		100%	98.3%	96.0%
Decision tree + RAM method		98.60%	98.3%	95.3%

## Data Availability

Not applicable.
